# Human vision is determined based on information theory

**DOI:** 10.1038/srep36038

**Published:** 2016-11-03

**Authors:** Alfonso Delgado-Bonal, Javier Martín-Torres

**Affiliations:** 1 Instituto Andaluz de Ciencias de la Tierra (CSIC-UGR), Avda. de Las Palmeras no. 4, Armilla, 18100, Granada, Spain; 2 Universidad de Salamanca, Instituto de Física Fundamental y Matemáticas, Pza de la Merced S/N, 37008, Salamanca, Spain; 3 Division of Space Technology, Department of Computer Science, Electrical and Space Engineering, Luleå University of Technology, Kiruna, Sweden

## Abstract

It is commonly accepted that the evolution of the human eye has been driven by the maximum intensity of the radiation emitted by the Sun. However, the interpretation of the surrounding environment is constrained not only by the amount of energy received but also by the information content of the radiation. Information is related to entropy rather than energy. The human brain follows Bayesian statistical inference for the interpretation of visual space. The maximization of information occurs in the process of maximizing the entropy. Here, we show that the photopic and scotopic vision absorption peaks in humans are determined not only by the intensity but also by the entropy of radiation. We suggest that through the course of evolution, the human eye has not adapted only to the maximum intensity or to the maximum information but to the optimal wavelength for obtaining information. On Earth, the optimal wavelengths for photopic and scotopic vision are 555 nm and 508 nm, respectively, as inferred experimentally. These optimal wavelengths are determined by the temperature of the star (in this case, the Sun) and by the atmospheric composition.

Vision is based on the absorption of light by photoreceptor cells in the eye. In humans, under luminous conditions, photopic vision mediated by cones is the dominant light-capture procedure, with the maximum absorption peak at approximately 555 nm. In low-light environments, scotopic vision mediated by rod cells dominates, with the maximum absorption occurring at 507 nm^1^. Our eyes use light to obtain information about our surroundings, a process that is not only about the perception of the environment by capturing energy but also about its interpretation^2^. The brain and cognitive functions are centred on learning through Bayesian inference^3^. The characteristics of human visual space are determined probabilistically^4^, and the interpretation of the signals to infer the environment starts in vision, following a Bayesian approach to obtain information about the environment^5^.

The absorption of light is the process of capturing photons: indivisible entities with a definite energy as a function of their wavelength, with a particular distribution along the electromagnetic spectrum depending on the temperature of the source. Nevertheless, radiation also contains entropy^6^, which follows a different distribution than the intensity. In the interpretation of the surroundings – the obtaining of information – through Bayesian statistics to improve the knowledge about the system, entropy plays a major role^7^.

Entropy is one of the most fundamental concepts in nature, and its domain extends beyond classical thermodynamics. The statistical definition of entropy proposed by Boltzmann – Gibbs entropy – has found extensive applications in the information theory developed by Shannon in the field of telecommunications^8^. Information theory shows the entropy as the average (expected) amount of information of a certain event. Maximizing the entropy is equivalent to maximizing the information content per observation. This theory is not limited to telecommunications, and it has been applied successfully to many other fields of research, including radiation^9^. Jaynes^10^ showed that Shannon’s mathematical theory of communication allows us to interpret the Boltzmann entropy density of a system as a measure of the information about the system that is not constrained in the distribution function^11^, i.e., it is possible to obtain information about the system from the entropy of radiation that is not available from the analysis of the energy.

According to information theory, the entropy of radiation determines the obtainable information from a radiation system, and photons of electromagnetic radiation are basically the carriers of information. In communication processes which function via radiation, information comes through non-interacting units of information (photons) at different wavelengths (*λ*); the total information can be described as the sum of the pieces of information, i.e., the total entropy is the sum of the entropies at each wavelength for incoherent radiation, 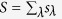
12, where *s*_*λ*_ is the entropy per wavelength. Entropy is a distribution concept, and it is not possible to talk about entropy of individual wavelengths if it is not compared with other wavelengths. The maximization of entropy is then the maximization of the entropy distribution, i.e. the Mode of the entropy function. If the available wavelengths for detecting radiation are limited, it is useful to identify those wavelengths where the entropy is maximal in order to maximize the obtainable information; the ability to choose wavelengths with more information is essential for an efficient design.

Planck derived the distribution function of the intensity for blackbody radiation based on Boltzmann’s statistical definition of entropy, in which the maximum is determined by Wien’s displacement law as a function of the temperature. He also provided an analytical expression of the entropy of radiation (entropy of non-interacting bosons), although the maximum of this distribution has remained undetermined until now. Here, we obtain a law for the maximum of the entropy of radiation similar to Wien’s displacement law by solving a transcendental equation (see supplementary material) which leads to *λT* = *b*_*entropy*_ = 3.00292 × 10^−3^ *mK*.

Understanding this equation in the context of information theory, the displacement law for the entropy of radiation presented here determines the wavelength at which the transference of information by radiation is maximized between a body at temperature T and an observer. The values of Wien’s constant for the energy (b_energy_ = 2.89777 · 10^−3 ^m K) and the entropy (b_entropy_ = 3.00292 · 10^−3 ^m K) are slightly different, meaning that the maximum for the two distributions occur at different wavelengths. The main conclusion is that the maximum of the intensity will always be at shorter wavelengths than the maximum of the entropy. Indeed, the ratio of the displacement laws of entropy and energy (Wien’s law) provides the “maximum entropy displacement law”, *λ*_*entropy*_ = *d*·*λ*_*energy*_, where *λ*_*entropy*_ is the wavelength at which the maximum of the spectral entropy is accomplished, *λ*_*energy*_ is the equivalent for the energy, and the coefficient 
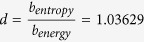
 is a dimensionless proportionality constant.

Figure 1 shows a schematic representation of the normalised spectra of the energy and the entropy for a blackbody at the average temperature of the surface of the Sun (5800 K). The maximum of the energy is observed at 500 nm, and the maximum of the entropy occurs at 518 nm. For wavelengths shorter than 500 nm, the energy spectrum is above the entropy spectrum, and for wavelengths larger than 518 nm, the situation is the opposite, with the entropy spectrum over the energy spectrum. Between the maxima of the two distributions, the product of the two distributions is maximized. The wavelength at which this is accomplished is determined only by the temperature, *λT* = *b*_*optimal*_ = 2.94865·10^−3^ m K, and this is presented in Fig. 2 (see supplementary material). For a 5800 K blackbody, this wavelength of *optimal information efficiency* is 508 nm.

From Figs 1 and 2, we see that, for a given temperature, photons at wavelengths larger than the maximum product will have more entropy (more information), but the intensity of the photons will be reduced. On the other hand, photons at shorter wavelengths will have more energy but a decreased content of information. Therefore, this function provides those values at which the content of information and energy per wavelength is optimized. It is neither the most energetic nor the most entropic, but it contains the best balance between energy and entropy. We have chosen this optimization rule by considering that energy and entropy are equally important in the considered environment. In this particular case, this equal weighting rule is applied because there is no reason to discriminate a magnitude versus the other.

The currently effective temperature of our sun (5800 K) is almost the same as in the Cambrian Explosion^13^, when it is believed that the first eyes appeared^14^ and continued to evolve. The radiation from the Sun passes through the atmosphere, interacting with molecules in processes such as scattering and absorption. We find that the maximum of the radiation is located at 547 nm, and the maximum of the entropy is reached at 564 nm (see Methods). The optimal wavelength, defined before as the wavelength where the product of the two distributions is maximized, occurs at 555 nm, exactly matching the photopic absorption peak. In situations in which radiation passes through the atmosphere without scattering, such as in late afternoon when the environment shifts to low light, the maximum of the product is located at 508 nm, almost identical to the scotopic vision absorption peak (507 nm).

These results suggest that for the optimal design of an instrument to detect and interpret the environment, the optimal wavelength will be the given by the proposed equation *λT* = *b*_*optimal*_. This suggests that the evolution of the human eye is optimized to look not for the maximum intensity of radiation or for the maximum information but for the optimal wavelength at which to obtain information about the environment.

There have been discussions addressing whether or not the eye’s evolution was determined by the Sun, with arguments that the maximum of Wien’s law in frequency is in the infrared instead of in the visible region. New studies show that the integration of the distribution functions to obtain the total energy available is independent on the variable^15^. As we are interested in the absorption peak, integrating in nm, we determine the maximum absorption of day light vision is at 555 nm, and the maximum absorption of low light vision is at 508 nm.

Here we have made use of the entropy of radiation to determine the optimal wavelength. Although it might be seen as a new approach, it is remarkable the fact that the entropy concept in information theory is commonly applied to computer vision problems. In those processes, which try to replicate our vision to identify persons, shapes or distances, the decisions are made based on the maximum entropy principle^16^. The entropy concept is not limited to the statistical mechanical understanding of the microscopic state of radiation, and information theory makes a different use of it. According to Jaynes^10^, “Information theory provides a constructive criterion for setting up probability distributions on the basis of partial knowledge, and leads to a type of statistical inference which is called the maximum-entropy estimate. It is the least biased estimate possible on the given information; i.e., it is maximally noncommittal with regard to missing information”.

In this paper, we rely upon the hypothesis that human vision was forced by the solar spectrum during the course of evolution and study it from a different perspective. We show that if vision was indeed forced, the entropy (and not only the energy) was of importance; it is a new analysis of the same hypothesis and we show the implications of it. There is, however, a question remaining which has not been addressed in the manuscript, which is why humans and no other living beings have those optimal absorption peaks. The questions arises from the original hypothesis in which this manuscript is based, i.e. vision was forced by the solar spectrum, and unfortunately there is not currently an explanation (nor physical or biological) of the different absorption peaks of the different species.

However, the inclusion of the entropy in the analysis could offer us a different perspective, which might be interesting to notice. In this manuscript, an equal weighting rule between the energy and the entropy was applied because there was no *a priori* reason to diminish the importance of one versus the other. In computer vision, for example, this rule is not applicable. In those analysis, the energy (size of the pixel) is constant and only the entropy changes (content of the pixel). In those situations, the energy is constant and the rule is reduced to the maximum entropy rule (see supplementary material), which is currently being applied.

The solar spectrum is not constant in wavelength, and therefore we have considered the energy function in the analysis. Different species might use energy and entropy differently, and the weighting rule should be modified accordingly. The vision of fishes is adapted to ultraviolet radiation, suggesting that under water the need of energetic photons overcomes the need of radiation information. Light comes only from a light pencil whereas the information about the environment can be inferred by other means (such as water currents or temperature). In those circumstances, the equal weighting rule is not applicable either. While this is not a validation of the hypothesis, this line of thought offers a new line of research which could lead to a better understating of the sight and the differences in vision between organisms from the point of view of the perception and interaction with the environment.

In this paper we do not analyse different species nor prove that vision was adapted to the solar spectrum. We only present here that human vision matches the defined optimal wavelength, and that the entropy analysis could provide more information which is currently being overlooked. The entropy of radiation is of the utmost importance in engineering and thermodynamics in areas such as heat transfer^17^ and climate sciences^18^, and it is essential for determining the maximum performance of solar energy conversion devices^19^ and the exergy of radiation^20^. Applying information theory to the radiation, we suggest that the evolution of human vision was forced by a follow-the-information rule. The determination of the maximum of the entropy of radiation allows us to identify the best wavelengths for sending information via radiation, which has implications not only for human eye evolution but also has far-reaching implications for instrument design, more effective telecommunication systems, astrobiology research or in general every system where information is transferred by photons.

## Methods

**Figure 1:**
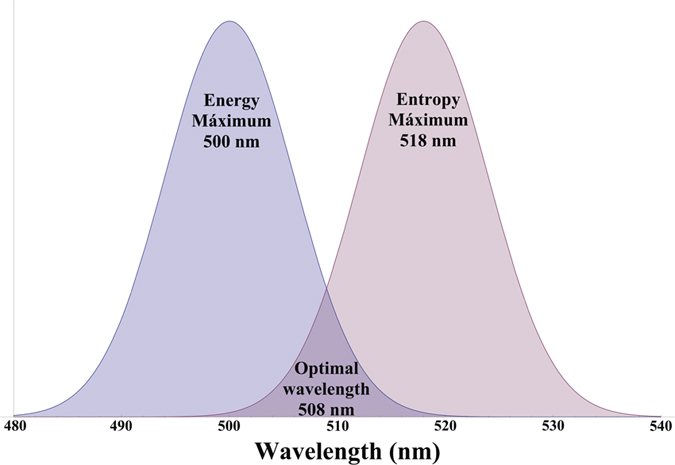
Optimal Information Wavelength. Schematic representation of the distributions of the energy and entropy of radiation for a blackbody at 5800 K. The maxima of the two distributions occur at different wavelengths, 500 nm for energy and 518 for entropy. The maximum information efficiency wavelength is defined as the position at which the product of both distributions is maximized, and corresponds in this case to 508 nm. The value of the wavelengths is dependent only on the temperature, and does not include atmospheric effects such as scattering (see supplementary material).

The distributions of the energy and entropy for a blackbody without atmospheric corrections are represented in Fig. 1. The optimal information wavelength for a blackbody at 5800 K corresponds to 508 nm, which matches the scotopic vision peak. On Earth, it corresponds to low light environment at sunset. The mathematical derivation is shown in the supplementary material. The transcendental equation is numerically solved to obtain the value of the associated Wien’s coefficient.

Radiation changes during its path through the atmosphere, interacting with molecules in absorption/emission or scattering processes. These interactions change the properties of the radiation that reaches the surface. We have used the radiative transfer model FUTBOLIN (FUll Transfer By Optimized LINe-by-line, freely available upon request)^21^ to calculate the transmission spectra reaching the surface. We have modelled the radiation at top of the atmosphere (TOA) as blackbody radiation at 5800 K, and analysed the Rayleigh scattering using the method proposed by Bodhaine *et al*.^22^. The radiation reaching the surface is then *I*_*surf*_ = *I*_*BB*_·*Tra*·*e*^*τ*^, where *I*_*BB*_ corresponds to the radiation emitted by the blackbody, Tra is the transmission spectra due to absorption and *τ* is the Rayleigh scattering component. The peak shifted to 555 nm, which matches the photopic vision peak. On Earth, the situation corresponds to full light environment at noon.

**Figure 2:**
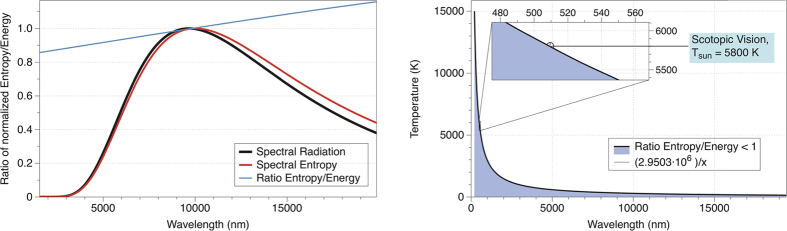
(left): Entropy content in radiation.The normalized distributions of energy and entropy are schematically represented for blackbody at 300 K. The entropy content in radiation ratio is not uniform, and it is unity for a certain value between the maxima of both distributions. The entropy content beyond this point is monotonically increasing. (right): Entropic regions and optimal information wavelength boundary. Entropic spectral regions as a function of the wavelength and temperature of the blackbody. As a consequence of the different location of the maxima, the spectra is divided in regions where normalized entropy is below the energy (blue region) or above the energy (white region). The separation of these regions is determined by the function *λT* = 2.94923 × 10^−3^ m K (see supplementary material). The region for 5800 K blackbody is zoomed in, corresponding to our Sun.

The study is not limited to the Sun and is evaluated for other blackbody temperatures, which would correspond to different types of stars. In Fig. 2 (right) we represent the two entropic regions of the spectra as a function of the temperature and wavelength. As the maxima is accomplished at different locations, the content of entropy in radiation per wavelength is different. Figure 2 (left) represents a normalized blackbody at 300 K; the energy distribution (black line) is over the entropy distribution (red line) until a certain wavelength dependent on the temperature. Beyond this point, the content of entropy in radiation (blue line) increases. The limiting function, value between the maxima of the energy and the entropy, is derived in the supplementary material.

**Figure 3:**
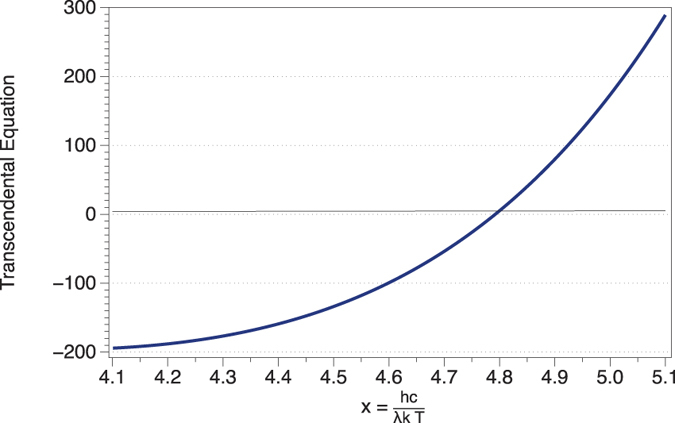
Wien’s peak for the entropy of radiation. The distribution of the entropy of radiation is different from the energy distribution. Its maximum (*dS*/*dλ* = 0) leads to a transcendental equation after the substitution 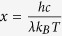 which is numerically solved: 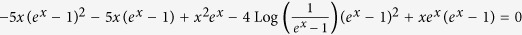. The solution gives x = 4.7912673578, and undoing the change of variable the relation *λT* = 3.00292 × 10^−3^ m K is obtained.

The solution of the entropy equation leads to a transcendental equation in the same way as Wien’s law does for energy. The solution of this equation is represented in Fig. 3. Mathematically, the condition of the maxima is imposed dS/dλ = 0, and the final equation is numerically solved.

Under other atmospheric conditions, such as the presence of clouds, the optimal wavelength would be shifted. Different types of clouds reshape the spectra in different ways, and it is not possible to find an optimal wavelength for these conditions. However, these situations are temporary and it is not likely that eyesight would be able to adapt to such conditions.

## Additional Information

**How to cite this article**: Delgado-Bonal, A. and Martín-Torres, J. Human vision is determined based on information theory. *Sci. Rep.* **6**, 36038; doi: 10.1038/srep36038 (2016).

**Publisher’s note**: Springer Nature remains neutral with regard to jurisdictional claims in published maps and institutional affiliations.

## Supplementary Material

Supplementary Information
