# Supplementary material for: Human vision is determined based on information theory
Source: Sci Rep. 2016 Nov 3;6:36038. doi: 10.1038/srep36038 (PMC5093619; doi:10.1038/srep36038)
Supplement: Supplementary Information [file srep36038-s1.pdf]

Human vision is determined based on information theory:

Supplementary Information

Alfonso Delgado-Bonal [1,2*] and F. Javier Martn Torres[1,3]

[1] Instituto Andaluz de Ciencias de la Tierra (CSIC-UGR), Avda. de Las Palmeras n 4, Armilla, 18100, Granada, Spain

[2] Universidad de Salamanca, Instituto de Física Fundamental y Matemáticas, Pza de la Merced S/N, 37008, Salamanca, Spain

[3] Division of Space Technology, Department of Computer Science, Electrical and Space Engineering, Luleå University of Technology, Kiruna, Sweden

* alfonso_delgado@usal.es

1 Supplementary information

Figure 3: Wien's displacement law of the entropy of radiation Using the statistical definition of entropy proposed by Boltzmann, Planck determined the analytical expression of the entropy of radiation (entropy of bosons):

$$S_\lambda = \frac{2kc}{\lambda^4}\left\{\left(1+\frac{\lambda^5 L_\lambda}{2hc}\right)\log\left(1+\frac{\lambda^5 L_\lambda}{2hc}\right)-\frac{\lambda^5 L_\lambda}{2hc}\log\frac{\lambda^5 L_\lambda}{2hc}\right\} \tag{1}$$

and afterwards derived the law that characterizes the behavior of the radiation intensity (Planck's law):

$$L_\lambda = \frac{2hc^2}{\lambda^5}\frac{1}{e^{\frac{hc}{k\lambda T}}-1} \tag{2}$$

Once the distribution of the radiation energy is known, is possible to derive the law that determines the maximum of energy for a given blackbody temperature, which is called Wien's displacement law. This law states that the spectral radiance of black body radiation per unit wavelength, peaks at the wavelength λ_{max} that is inversely proportional to temperature:

$$\lambda_{\text{max}} = \frac{b}{T} \tag{3}$$

where T is the absolute temperature of the body in Kelvin and b is a constant of proportionality (called Wien's constant) equal to 2.8977721(26)$\times 10^{-3}$ m K. The constant b was determined experimentally, firstly by Lummer and Pringsheim and later by Paschen, and was theoretically obtained by Planck once its distribution law was formulated. It is worth noticing that Wien proposed the law that carries his name before Planck obtained the spectral distribution.

Wien's law determines the wavelength of the maximum emission of radiation by a blackbody, but it should not be confused with the maximum information. Following the reasoning proposed by Planck in the Part II of this book [Planck, 1914], the Wien's displacement law should not be restricted to the spectral intensity of radiation. Moreover, the spectral entropy of radiation will follow a similar law, but the value of the constant does not have to be the same.

Equation 20 can be rewritten as:

$$S_\lambda = \frac{2kc}{\lambda^4}\left\{(1+x)\log(1+x) - x\log x\right\} \tag{4}$$

where $x = \frac{L_\lambda \cdot \lambda^5}{2hc^2} = \frac{1}{e^{hc/\lambda kT} - 1}$. It can be converted by simple arithmetic into:

$$S_\lambda = \frac{2hc^2}{T\lambda^5} + \frac{1}{T}L_\lambda + \frac{2kc}{\lambda^4}\cdot\log\left(\frac{1}{e^{\frac{hc}{\lambda kT}} - 1}\right) \tag{5}$$

In the maximum of the function we have the condition is $\frac{dS_\lambda}{d\lambda}$ = 0, which leads to:

$$\begin{aligned}\frac{dS_\lambda}{d\lambda} =& \frac{-10hc^2}{\lambda^6 T} + \frac{1}{T}\frac{-10hc^2}{\left(e^{\frac{hc}{\lambda kT}} - 1\right)\cdot\lambda^6} + \frac{1}{T}\cdot\frac{2h^2c^3}{\lambda^7 kT}\cdot\frac{e^{\frac{hc}{\lambda kT}}}{\left(e^{\frac{hc}{\lambda kT}} - 1\right)^2} \\ &+ \frac{-8kc}{\lambda^5}\cdot\log\left(\frac{1}{e^{\frac{hc}{\lambda kT}} - 1}\right) + \frac{2hc^2}{\lambda^6 T}\frac{e^{\frac{hc}{\lambda kT}}}{e^{\frac{hc}{\lambda kT}} - 1} = 0\end{aligned} \tag{6}$$

Looking for common denominator, $\lambda^7 T^2\left(e^{\frac{hc}{\lambda kT}} - 1\right)^2$:

$$\begin{aligned}\frac{dS_\lambda}{d\lambda} =& \frac{-10hc^2\lambda T\left(e^{\frac{hc}{\lambda kT}}-1\right)^2}{\lambda^7T^2\left(e^{\frac{hc}{\lambda kT}}-1\right)^2} + \frac{-10hc^2\lambda T\left(e^{\frac{hc}{\lambda kT}}-1\right)}{\lambda^7T^2\left(e^{\frac{hc}{\lambda kT}}-1\right)^2} \\ &+ \frac{2h^2c^3e^{\frac{hc}{\lambda kT}}}{\lambda^7T^2\left(e^{\frac{hc}{\lambda kT}}-1\right)^2} - \frac{8kc\cdot\log\left(\frac{1}{e^{\frac{hc}{\lambda kT}}-1}\right)\lambda^2T^2\left(e^{\frac{hc}{\lambda kT}}-1\right)^2}{\lambda^7T^2\left(e^{\frac{hc}{\lambda kT}}-1\right)^2} \\ &+ \frac{2hc^2e^{\frac{hc}{\lambda kT}}\lambda T\left(e^{\frac{hc}{\lambda kT}}-1\right)}{\lambda^7T^2\left(e^{\frac{hc}{\lambda kT}}-1\right)^2} = 0\end{aligned} \tag{7}$$

The numerator must be zero in the previous equation in order for the entropy to be a maximum:

$$\begin{aligned}\frac{dS_\lambda}{d\lambda} =& -10hc^2\lambda T\left(e^{\frac{hc}{\lambda kT}}-1\right)^2 + -10hc^2\lambda T\left(e^{\frac{hc}{\lambda kT}}-1\right) + 2h^2c^3e^{\frac{hc}{\lambda kT}} \\ &- 8kc\cdot\log\left(\frac{1}{e^{\frac{hc}{\lambda kT}}-1}\right)\lambda^2T^2\left(e^{\frac{hc}{\lambda kT}}-1\right)^2 \\ &+ 2hc^2e^{\frac{hc}{\lambda kT}}\lambda T\left(e^{\frac{hc}{\lambda kT}}-1\right) = 0\end{aligned} \tag{8}$$

Using the transformation x = $\frac{hc}{\lambda kT} \Rightarrow \lambda T = \frac{hc}{kx}$:

$$\begin{aligned}\frac{dS_\lambda}{d\lambda} =& -\frac{10h^2c^3\left(e^x-1\right)^2}{kx} - \frac{10h^2c^3\left(e^x-1\right)}{kx} + \frac{2h^2c^3e^x}{k} \\ &- 8kc\cdot\log\left(\frac{1}{e^x-1}\right)\frac{h^2c^2}{k^2x^2}\left(e^x-1\right)^2 + 2hc^2e^x\frac{hc}{kx}\left(e^x-1\right) \\ =&0\end{aligned} \tag{9}$$

Removing common factor $\frac{2h^2c^3}{k}$:

$$\frac{dS_\lambda}{d\lambda} = -\frac{5\left(e^x-1\right)^2}{x} - \frac{5\left(e^x-1\right)}{x} + e^x - 4\log\left(\frac{1}{e^x-1}\right)\frac{\left(e^x-1\right)^2}{x^2} + \frac{e^x\left(e^x-1\right)}{x} = 0 \tag{10}$$

and multiplying by x^2:

$$\frac{dS_\lambda}{d\lambda} = -5x\left(e^x-1\right)^2 - 5x\left(e^x-1\right) + x^2\cdot e^x - 4\log\left(\frac{1}{e^x-1}\right)\left(e^x-1\right)^2 + x\cdot e^x\left(e^x-1\right) = 0 \tag{11}$$

we obtain a transcendental equation which cannot be solved analytically but that can be solved numerically (Figure 3). The solution gives x = 4.7912673578 and reversing the x variable:

$$x = \frac{hc}{\lambda kT} \Rightarrow \lambda T = \frac{hc}{k\cdot 4.7912673578} = 3.00292\times 10^{-3}\text{m K} \tag{12}$$

which is the Wien's displacement law of the entropy of radiation.

Figure 2: Function of normalized ratio of entropy to energy equal to unity The entropy content in radiation is not uniformly distributed along the spectra. The different location of the maxima and the shape of the spectra divides the space in two entropic regions. The curve that limits these regions is characterized for having a value of the ratio of normalized entropy to energy equal to the unity. The curve is verifies the equation:

$$\frac{\frac{S_\lambda}{S_{\lambda,max}}}{\frac{L_\lambda}{L_{\lambda,max}}} = 1 \tag{13}$$

Where S_λ is given by Equation 20, L_λ by Equation 21, and $S_{\lambda,max}$ and $L_{\lambda,max}$ are the maximum of the entropy and the energy respectively. The relation is rewritten as:

$$S_\lambda \cdot L_{\lambda,max} = S_{\lambda,max} \cdot S_\lambda \tag{14}$$

As the wavelength of the maximum of the entropy and the energy are determined by the Wien's displacement laws described in this paper, we can use the relations $\lambda_{\text{max,energy}} \cdot T = b_{\text{max,energy}}$ and $\lambda_{\text{max,entropy}} \cdot T = b_{\text{max,entropy}}$ in Equations 20 and 21 to determine $S_{\lambda,max}$ and $L_{\lambda,max}$. Using the expression of the entropy of Equation 5, we have:

$$S_{\lambda,\text{max}} = \frac{2hc^2 \cdot T^5}{T \cdot b_{\text{entropy}}^5} + \frac{1}{T}\frac{2hc^2}{b_{\text{entropy}}^5}\frac{T^5}{e^{\frac{hc}{kb_{\text{entropy}}}} - 1} + \frac{2kc}{b_{\text{entropy}}^4} \cdot T^4 \cdot \log\left(\frac{1}{e^{\frac{hc}{kb_{\text{entropy}}}} - 1}\right) \tag{15}$$

$$L_{\lambda,\text{max}} = \frac{2hc^2}{b_{\text{energy}}^5}\frac{T^5}{e^{\frac{hc}{kb_{\text{energy}}}} - 1} \tag{16}$$

For simplicity, we call $c_1 = 2hc^2$, $c_2 = hc/k$ and $c_3 = 2kc$. The relation described in Equation 14 leads to the equation:

$$\frac{c_1}{T\cdot\lambda^5}\cdot\frac{c_1}{b_{\text{energy}}^5}\frac{T^5}{\left(e^{c_2/b_{\text{energy}}}-1\right)}+\frac{1}{T}\frac{c_1}{\lambda^5}\frac{1}{\left(e^{c_2/\lambda T}-1\right)}\frac{c_1}{b_{\text{energy}}^5}\frac{T^5}{\left(e^{c_2/b_{\text{energy}}}-1\right)}$$
$$+\frac{c_3}{\lambda^4}\cdot\log\left(\frac{1}{e^{c_2/\lambda T}-1}\right)\cdot\frac{c_1}{b_{\text{energy}}}\frac{T^5}{\left(e^{c_2/b_{\text{energy}}}-1\right)}=$$
$$\frac{c_1}{T}\frac{T^5}{b_{\text{entropy}}^5}\cdot\frac{c_1}{\lambda^5}\frac{1}{\left(e^{c_2/\lambda T}-1\right)}+\frac{1}{T}\frac{c_1}{b_{\text{entropy}}^5}\frac{T^5}{\left(e^{c_2/b_{\text{entropy}}}-1\right)}\frac{c_1}{\lambda^5}\frac{1}{\left(e^{c_2/\lambda T}-1\right)}$$
$$+\frac{c_3}{b_{\text{entropy}}^4}\cdot T^4\cdot\log\left(\frac{1}{e^{c_2/b_{\text{entropy}}}-1}\right)\cdot\frac{c_1}{\lambda^5}\frac{1}{\left(e^{c_2/\lambda T}-1\right)} \qquad (17)$$

It is easy to prove that removing $\frac{T^4}{\lambda^5}$, calling $x=\frac{c_2}{\lambda T}$ and multiplying both sides by (e^x-1), the equation is reduced to:

$$e^x+\frac{e^x-1}{x}\cdot\log\left(\frac{1}{e^x-1}\right)=\left(\frac{b_{\text{energy}}}{b_{\text{entropy}}}\right)^5\cdot\left(e^{\frac{hc}{kb_{\text{energy}}}}-1\right)\cdot\left\{1+\frac{1}{e^{\frac{hc}{kb_{\text{entropy}}}}-1}+\frac{kb_{\text{entropy}}}{hc}\cdot\log\left(\frac{1}{e^{\frac{hc}{kb_{\text{entropy}}}}-1}\right)\right\} \qquad (18)$$

that is a transcendental equation with numerical solution x = 4.878482. Undoing the change of variable, we have:

$$\lambda T=\frac{hc}{k\cdot 4.8784820}=2.94923\cdot 10^{-3}\text{m K} \qquad (19)$$

Figure 1: Optimal wavelength: maximum product As explained in the main text, the product of energy and entropy is maximized in a point between the maxima of both distributions. The equation that determines the optimal wavelength is derived as the maxima of the product of the two distributions. As seen before, the distributions of entropy and energy are respectively:

$$S_\lambda = \frac{2kc}{\lambda^4}\left\{\left(1+\frac{\lambda^5 L_\lambda}{2hc}\right)\log\left(1+\frac{\lambda^5 L_\lambda}{2hc}\right)-\frac{\lambda^5 L_\lambda}{2hc}\log\frac{\lambda^5 L_\lambda}{2hc}\right\} \tag{20}$$

$$L_\lambda = \frac{2hc^2}{\lambda^5}\frac{1}{e^{\frac{hc}{k\lambda T}}-1} \tag{21}$$

and their derivates:

$$\begin{aligned}\frac{dS_\lambda}{d\lambda} =& \frac{-10hc^2}{\lambda^6 T}+\frac{1}{T}\frac{-10hc^2}{\left(e^{\frac{hc}{\lambda kT}}-1\right)\cdot\lambda^6}+\frac{1}{T}\cdot\frac{2h^2c^3}{\lambda^7 kT}\cdot\frac{e^{\frac{hc}{\lambda kT}}}{\left(e^{\frac{hc}{\lambda kT}}-1\right)^2}\\ &+\frac{-8kc}{\lambda^5}\cdot\log\left(\frac{1}{e^{\frac{hc}{\lambda kT}}-1}\right)+\frac{2hc^2}{\lambda^6 T}\frac{e^{\frac{hc}{\lambda kT}}}{e^{\frac{hc}{\lambda kT}}-1}\end{aligned} \tag{22}$$

$$\frac{dL_\lambda}{d\lambda} = \frac{-10hc^2}{\left(e^{\frac{hc}{\lambda kT}}-1\right)\cdot\lambda^6}+\frac{2h^2c^3}{\lambda^7 kT}\cdot\frac{e^{\frac{hc}{\lambda kT}}}{\left(e^{\frac{hc}{\lambda kT}}-1\right)^2} \tag{23}$$

The maximum product is accomplished when $\frac{d}{d\lambda}(S_\lambda \cdot L_\lambda)=0$, i.e., $\frac{dS_\lambda}{d\lambda}\cdot L_\lambda+\frac{dL_\lambda}{d\lambda}\cdot S_\lambda = 0$.

Using the previous equations, removing the common factors $\lambda^{12}T^2k(e^{\frac{hc}{\lambda T}}-1)^3$, and hc^3, and doing the change of variable $x=\frac{hc}{\lambda kT}$, the equation is transformed to:

$$\begin{aligned}&-\frac{10}{x}(e^x-1)^2-\frac{10}{x}(e^x-1)+2e^x-\frac{9}{x^2}(e^x-1)^2\log\left(\frac{1}{e^x-1}\right)+\\ &\frac{e^x}{x}(e^x-1)+e^x(e^x-1)+\frac{e^x}{x}(e^x-1)\log\left(\frac{1}{e^x-1}\right)=0\end{aligned} \tag{24}$$

which numerically solved, gives the value $x = 4.8794390856$. Undoing the change of variable, we obtain $\lambda T = 2.94865 \cdot 10^{-3}$ m K.

In those situations where the equal weighting rule cannot be applied, the optimal function would have a different value. For example, in computer vision where the energy is constant, the optimal wavelength is determined by $\frac{d}{d\lambda}(S_\lambda \cdot L_\lambda) = L_\lambda \frac{d}{d\lambda}(S_\lambda) = 0$, i.e. it is reduced to the classical maximum entropy rule $\frac{dS_\lambda}{d\lambda} = 0$.
